# Molecular identification, incidence and phylogenetic analysis of seven viruses infecting garlic in Ethiopia

**DOI:** 10.1007/s10658-019-01760-9

**Published:** 2019-05-15

**Authors:** A. D. Abraham, D. B. Kidanemariam, T. A. Holton

**Affiliations:** 1 Department of Biotechnology, Addis Ababa Science and Technology University, P.O. Box 16417, Addis Ababa, Ethiopia; 2 National Agricultural Biotechnology Research Centre, Ethiopian Institute of Agricultural Research, P.O. Box 2003, Addis Ababa, Ethiopia; 3 Biosciences eastern and central Africa-International Livestock Research Institute (BecA-ILRI) Hub, P.O. Box 30709, Nairobi, Kenya

**Keywords:** Allexiviruses, *Allium*, Carlaviruses, IYSV, Potyviruses, Tospovirus

## Abstract

Little information exists on the type and incidence of viruses infecting garlic (*Allium sativum L*) in Ethiopia. Attempts were made to identify the viruses using molecular techniques from 95 composite leaf samples collected from 44 farmers’ fields and 51 germplasm accessions. Reverse transcription (RT-) PCR using genus and/or virus specific primers was used to amplify partial genome sequences of potyviruses, allexiviruses, carlaviruses and a tospovirus followed by sequencing of PCR products. Results indicated that ~73.7% of the samples are infected with at least one virus. *Onion yellow dwarf virus* (OYDV, genus *Potyvirus*, family *Potyviridae*) is the most common virus detected followed by *Garlic virus C* (genus *Allexivirus*) and *Shallot latent virus* (SLV, genus *Carlavirus*). Other viruses detected at lower frequency include *Garlic virus X* and *Garlic virus D* (genus *Allexivirus*), *Leek yellow stripe virus* (genus *Potyvirus*) and *Iris yellow spot virus* (IYSV, *genus Tospovirus*). Mixed infection of two or more viruses was detected in 65.7% of the samples. Phylogenetic analysis suggested that the different viruses may have been introduced to Ethiopia from Europe or Asia. This is the first report of *Garlic virus X, Garlic virus D*, IYSV and SLV in garlic in Ethiopia. The high incidence of OYDV and IYSV which cause severe yield loss alone or in mixed infection with allexiviruses and carlaviruses is a cause of concern to growers.

## Introduction

Garlic (*Allium sativum L*.) is a spicy vegetable crop widely cultivated throughout the world. In Ethiopia, the crop is grown as a major vegetable in an estimated area coverage of 19,000 ha in 2016 (CSA 2018). Diseases caused by viruses are among the major production constraints of the crop globally causing yield loss of up to 50% (Fujisawa 1989; Lot et al. 1998; Takaichi et al. 1998; Walkey and Antil 1989). The main disease symptoms on infected plants include reduction in plant vigor, mosaic and leaf striping, and leaf yellowing and deformations. Worldwide, several viruses have been known to naturally infect garlic, often in complex mixtures. Common among these are members of the genus *Potyvirus namely Onion yellow dwarf virus* (OYDV), *Leek yellow stripe virus* (LYSV), and *Shallot yellow stripe virus* (SYSV) and of the genus *Carlavirus namely Garlic common latent virus* (GarCLV) and *Shallot latent virus* (SLV, synonym: *Garlic latent virus*, GLV), all of which are aphid-transmitted (Fajardo et al. 2001; Takaichi et al. 1998). In addition, garlic is infected by several virus species belonging to the genus *Allexivirus* in family *Alphaflexiviridae*. Allexiviruses are known to be mite-transmitted and include *Garlic virus A* (GarVA), -B (GarV-B), -C (GarV-C), and –D (GarV-D), −E (GarV-E) and –X (GarV-X) and *Shallot virus X* (ShVX) (Chen et al. 2004; Dovas and Vovlas 2003; Lee et al. 2007; Park et al. 2005). Allexiviruses and carlaviruses often show latent infection in garlic in single infection while more severe diseases resulting in drastic reduction in yield can result from multiple infection (Cafrune et al. 2006; Lot et al. 1998; Paludan 1980). Recently, *Iris yellow spot virus* (IYSV), a member of the genus Tospovirus transmitted by thrips, is also reported to infect garlic in many areas of the world (Gawande et al. 2010; Gent et al. 2006; Karavina et al. 2016). The low average yield of garlic in Ethiopia is attributed to a number of factors among which viral diseases are suspected to take a considerable share. This is believed to be linked to the vegetative mode of propagation of the crop materials often leading to a gradual build of virus in subsequent generations (Conci et al. 2003).

Managing viral diseases in crops usually requires a thorough understanding of the identity, relative importance and distribution of the viruses. Currently, there is little information on viruses infecting garlic in Ethiopia. The only systematic garlic virus study in the country todate was based on serological test using ELISA (Jemal et al. 2015) and indicated the association of four viruses with the crop: two potyviruses (OYDV and LYSV) and two allexiviruses (GarV-B and -C). In that study however, many of the viruses known to infect garlic were not investigated, largely because specific reagent antibodies are not available. The absence of adequate information on virus diseases of garlic crops in Ethiopia has hampered efforts to devise suitable management options. Molecular method particularly reverse transcription polymerase chain reaction (RT-PCR), often followed by sequencing of certain regions of viral genome is considered better and more accurate alternative of virus identification than more commonly used serological methods (Chen et al. 2004; Lunello et al. 2005). This is mainly because of inherent drawbacks of serological tests which include the unavailability of reagent antisera to some known viruses, the cross reaction of some antisera to heterologous viruses and lesser sensitivity of serological reactions compared to PCR-based techniques. Subsequently, a number of generic and virus specific primers that help in specific identification of viruses infecting *Allium* crops by RT-PCR have been made available (Arya et al. 2006; Chen et al. 2004; Dovas et al. 2001; Takaichi et al. 2001; Tsuneyoshi et al. 1998a, 1998b; Zheng et al. 2010).

In this paper, RT-PCR and Sanger sequencing were employed to more accurately identify viruses infecting garlic in Ethiopia and determine their relative incidence and phylogenetic relationship to previously reported virus isolates elsewhere.

## Materials and method

### Sample collection, RNA extraction and cDNA synthesis

Garlic leaf samples (*n* = 44) were collected in May 2013 from 44 farmers’ fields (area ranging from 0.5 to 2.0 ha for each field) representing eight administrative zones (Table S1) from various major growing locations in Ethiopia and 51 germplasm accessions maintained at Debre Zeit Agricultural Research Center (DZARC), Oromia, Ethiopia (Table S1). Garlic crops consisting of both local garlic land races and three improved varieties (Bishoftu Nech, Tseday and Kuriftu) commonly cultivated in Ethiopia were surveyed just before flowering stage. Fields were selected in the main garlic growing areas of the different zones by travelling on the main roads and stopping in every 5–10 km wherever the crop is found. From each field or germplasm accession plot, leaf samples comprising both symptomatic and asymptomatic ones were collected by randomly harvesting leaves from 8 to 10 plants, making a composite sample, by moving in zigzag pattern along the fields and accession plots. Each sample was separately desiccated over silica-gel kept in air tight falcon tube and transported to BecA-ILRI Hub, Nairobi, Kenya for in vitro laboratory analysis. RNA was extracted using ZR Plant RNA MiniPrep kit (Zymo Research, USA). cDNA was synthesized with Maxima First strand cDNA synthesis kit, (Thermo Scientific, USA) as per the manufacturer’s recommendation with the modification that reverse primers employed for respective PCR amplification (Table 1) were included in the reaction mixture.

**Table 1:** PCR primers used for amplification of part of the genome of garlic viruses. Note that all genus-specific primers and some speciesspecific primers are degenerate. The letters F and R in front of the primer codes designate forward and reverse primers, respectively

Target Genus/Virus species	Primer Code	Primer Sequence (5′ to 3′)	Reference
*Potyvirus*	NIB2-F NIB3-R	GTITGYGTIGAYGAYTTYAAYAA TCIACIACIGTIGAIGGYTGNCC	Zheng et al. 2010
*Allexivirus*	CP + F NABP—R	TGGRCXTGCTACCACAAYGG CCYTTCAGCATATAGCTTAGC	Chen et al. 2004
*Carlavirus*	pCAR-1F PC-R4-R	ATGCCXCTXAXXCCXCC ACCGATTCAACTGGAAGAATTCGCGG	Tsuneyoshi et al. 1998b
*Onion yellow dwarf virus*	OYDV-F OYDV-R	ATAGCAGAAACAGCTCTTA GTCTCYGTAATTCACGC	Arya et al. 2006
*Leek yellow stripe virus*	GV2 -2 L1-F GV22R1-R	GGGTTCTTGAACAAGCACCG GATGGTGCATCCGTGCATTC	Takaichi et al. 1998
*Shallot yellow stripe virus*	SYS-UP-F SYSDW-R	GCAGGATCCAACACCRAGTTATGTGTC TTCGGATCCATRTGAGCTTCCTTCGC	Van der Vlugt et al. 1999
*Garlic common latent virus*	CAR-CP3-F CAR-V3-R	GTATGCAACTTAAATATAGCACGC GCTAGACATTGGTAGCCTTAGG	Tsuneyoshi et al. 1998
*Shallot latent virus*	SLV-F SLV-R	GTGGTNTGGAATTAC CAACATCGATTYTCTC	Majumder et al. 2008
*Iris yellow spot virus*	IYSV-F, IYSV-R	TAGGGTGAAACCGTCAGAAA TGTCTTGTAAATGCCTGCTC	Rafizadeh et al. 2012

### PCR and sequence analysis

For PCR amplification, AccuPower Ready to use PCR PreMix (Bioneer, USA) was used in a total reaction volume of 20 μl with cDNA as a template. Degenerate genus specific PCR primer pairs previously reported to amplify different potyviruses, allexiviruses and carlaviruses were used (Table 1). In addition, for viruses for which species-specific primers were available (OYDV, LYSV, SYSV), cDNAs synthesized using reverse primers from selected samples that were PCR positive with degenerate primers were screened with PCR using specific primers (Table 1). SLV and IYSV sequences were directly amplified by PCR from cDNA templates generated garlic leaf samples using specific primers (Table 1). A total of 5–50 ng cDNA (1 μl), 10 ρmole (1 μl) of each primers and 17 μl of nuclease free water was added to AccuPower Ready to use PCR PreMix lyophilized pellet. The pellet was dissolved by vortexing and spinning down the mix and the PCR was carried out following the PCR-cycling condition reported for each primer pairs. PCR products (5 μl) were separated and visualized by electrophoresis through 1.5% agarose gel. Upon successful amplification, the remaining PCR products (15 μl) were purified using GeneJet PCR purification kit (Thermo Scientific, USA) and submitted to BecA-ILRI Hub Segolip unit for Sanger sequencing.

Sequencing data were processed and analyzed using Geneious v11.0.2 (Biomatters, New Zealand) computer software and aligned with all known garlic viruses on the NCBI database using BLASTn available on the NCBI website (http://blast.ncbi.nlm.nih.gov/Blast.cgi). Virus sequences were further aligned and analyzed by the ClustalW using BioEdit sequence alignment editor program version 7 ( h t tp ://www.mbio.ncsu.edu/BioEdit/bioedit.html). Phylogenetic trees were constructed from ClustalW-aligned sequences on MEGA-X (http://www.megasoftware.net/mega.php), using the Maximum-Likelihood method with 1000 bootstrap replications. Pairwise sequence comparisons (PASC) were carried out on aligned sequences using Geneious v11.0.2 (Biomatters, New Zealand) computer software. To assess possible recombination events, sequences from the different virus groups from this study together with representative isolates from the NCBI database were analyzed using the RDP4 program (with embedded RDP, GENECONV, Bootscan, MaxChi, Chimaera, 3Seq and SiScan tools) (Martin et al. 2015). Only recombination events with a Bonferroni-corrected *P* value of <0.05 were considered credible while recombination signals labelled by RDP4 as potentially attributed to evolutionary processes other than recombination were disregarded.

## Results

### Virus incidence in fields and germplasm accessions

Virus-like symptoms such as leaf striping, mild mosaic and yellowing were observed in only few garlic fields during the survey and no clear virus-like symptoms were encountered in most fields.

From the total of 95 composite samples tested, 70 (73.7%) were infected with at least one virus with mixed infection of two or more viruses detected in 46 (65.7%) of the infected samples. Of the 44 composite samples each collected from the 44 farmer’s fields and tested by RTPCR for the different viruses, 33 (75%) were found to be infected with at least one virus whereas the remaining 11 (25%) did not test positive to any of the viruses screened in this study. Of those fields with virus infection, 11 (33.3%) had single infection, 15 (44.4%) had mixed infection of two viruses, whereas infection from three viruses is recorded in seven (~22.2%). None was infected with four or more viruses. In all the eight zones where samples were collected, different viruses were detected and there was no clear association of one virus or the other with specific administrative zone of the country. On the other hand, of 51 garlic germplasm accessions screened, 37 (~72.5%) were infected by at least one virus. Double infection was found in 11 (~29.7%) of the accessions and infection with three or more viruses was detected in the remaining 11 (~29.7%) of accessions screened. A total of 14 (~27.5%) of the accessions was found to be free from all the viruses tested in this study.

### Identification of potyviruses

A total of 25 samples, each representing a farmer’s field, and 21 of germplasm accessions were tested positive by RT-PCR using the generic potyvirus primers pairs targeting the NIb-coding region (Table S1) giving the product of the expected size (350 bp). Out of these 46 amplicons, sequence information was obtained for 40 samples representing the different zones. These sequences were subjected to BLASTn analysis of which 33 isolates showed highest nucleotide identity (84–89%) to a range of OYDV isolates, while the remaining seven isolates showed the highest nucleotide identity (82–87%) to different LYSV isolates. Subsequently, representative samples positive for potyvirus degenerate RT-PCR were subjected to RT-PCR using OYDV, LYSV and SYSV specific primers targeting coat protein (CP)-coding region. Virus-specific RT-PCR amplicon of the expected size were only obtained for OYDV and LYSV but not for SYSV. It was thus confirmed that of the 40 potyvirus positive samples, 33 samples contained OYDV, seven contained LYSV and none was positive for SYSV.

BLASTn analysis of the CP-coding nucleotide sequences of OYDV isolates from Ethiopia revealed 85–87% identity to OYDV isolate-72 from Poland (KF862684). Similarly, BLASTn analysis of the CP coding nucleotide sequence of LYSV showed 80–87% similarity to a range of LYSV from Latin America including isolates from Brazil (KP258216) and Mexico (KF597283), as well as isolates from Europe (Italy GQ475418 and Spain-HQ918255), Israel (AF071525) and Japan (AB194632).

### Identification of Allexiviruses

The allexivirus degenerate primer pair targeting the CP-coding region successfully amplified products of the expected band size of ~750 bp from a total of 41 samples tested. PCR products from all these samples were purified and sequenced. BLASTn analysis revealed that 31 samples are infected with GarV-C with 81–97% nucleotide identity to arange of GarVC isolates from Czech Republic and Sudan, making this virus the most common allexivirus in Ethiopian garlic crops. Eight samples showed the highest nucleotide identity of 85–96% to an Australian GarV-X isolate-SW3 (JQ807994), while the remaining two isolates (Et10 and Et20 GenBank accession number MH794550 and MH794555) showed the highest nucleotide identity of 83% to GarV-D isolate1058.3 from Czech Republic (JX682868). None of the amplicons generated by degenerate allexivirus primers gave sequences of ShVX, GarV–A and –B suggesting that these viruses are absent in our samples.

### Identification of carlaviruses

The reported generic carlavirus primer pair pCAR-1/ PC-R4 (Table 1) targeting the CP-coding region failed to amplify any virus RNA from our samples in repeated attempts. Therefore, samples were subjected to RT-PCR using primers reported to amplify two common carlaviruses infecting *Allium* crops namely *Garlic common latent virus* (GarCLV) and *Shallot latent virus* (SLV) (Table 1) targeting their CP-coding region. However, our attempts to amplify GarCLV using primers reported to be specific for this virus was not successful. On the other hand, an amplicon of the expected size (~300 bp) was obtained from 39 samples in RT-PCR using virus specific SLV primers. Isolates from eight representative samples were sequenced and BLASTn analysis revealed that SLV isolates from Ethiopia have highest nucleotide identity of 81–84% to GLV isolates from Poland, South Korea, and China.

### Identification of IYSV

By RT-PCR using IYSV specific primers (Table 1), a fragment of the expected band size (~180 bp) was obtained from seven samples (Table S1). PCR fragments were purified and sequenced. BLASTn analysis indicated that IYSV isolates from Ethiopia have the highest nucleotide identity of 98% to IYSV isolate from Sri Lanka (GU901211).

### Phylogenetic analysis, pairwise sequence comparison (PASC) and recombination analysis

Phylogenetic analysis and PASC were carried out to determine the evolutionary relationship of the different garlic viruses identified in this study. Since most isolates of the same virus species sequenced in this study showed high nucleotide sequence identity (data not shown), only representative ones were used in the phylogenetic analysis.

**Fig. 1:**
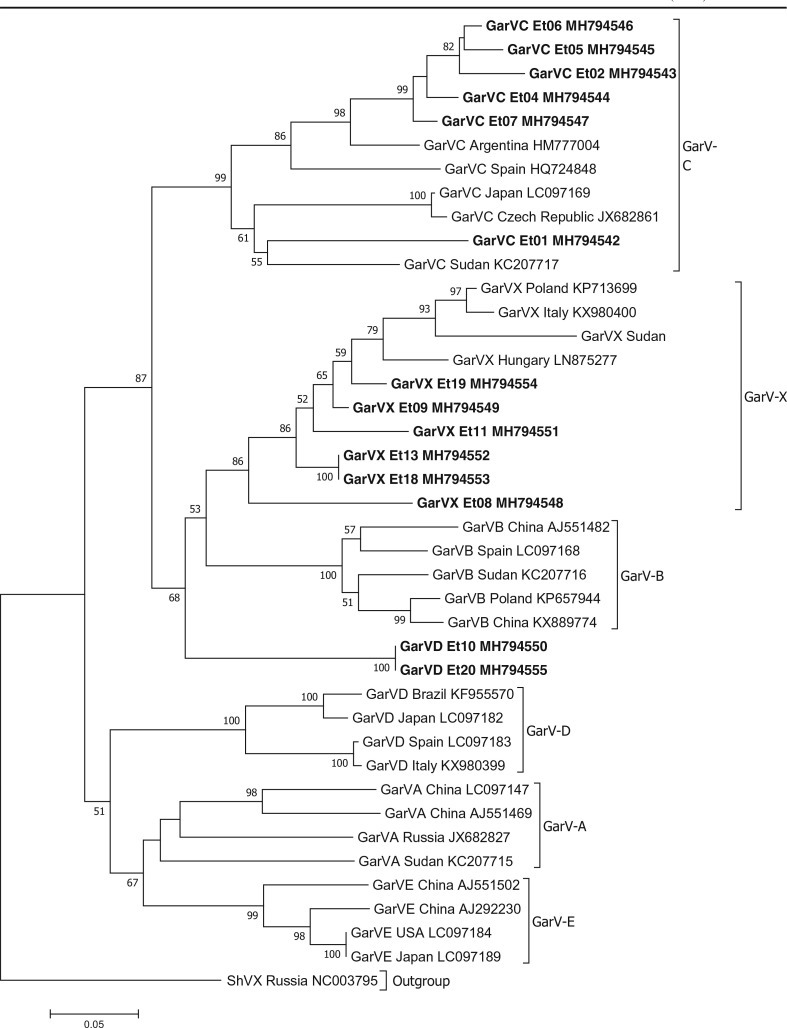
Phylogenetic tree based on nucleotide sequences showing the relationship of the different allexivirus isolates identified in this study with selected allexivirus sequences in the GenBank. Shallot virus X (ShVX) is used as an outgroup. Only bootstrap values greater than 50% are shown and the scale bar indicates substitutions per site. Isolates from this study are highlighted in bold

Phylogenetic analysis showed that allexiviruses sequences formed six different clades each containing the different allexivirus species as expected (Fig. 1). Five of the six GarV-C isolates from Ethiopia (Et02, 04, 05, 06 and 07 GenBank accession number MH794543, MH794544, MH794545, MH794546 and MH794547, respectively) clustered together with GarV-C isolates from Argentina (HM777004) and Spain (HQ724848) (Fig. 1) supported with high level of bootstrap value. On the other hand, one GarV-C isolate (Et01, GenBank MH794542) grouped together with GarV-C isolate from Sudan (KC207717) suggesting its clear divergence from the other GarV-C isolates from Ethiopia. In a cluster consisting of GarV-X sequences, all the isolates from Ethiopia clustered together, with isolate Et08 (GenBank accession number MH794548) appearing to be ancestral to the others (Fig. 1). GarV-X isolate from Hungary (GenBank accession number LN875277) has a closest genetic distance to the Ethiopian isolate whereas the isolate from Sudan, a country geographically closest to Ethiopia has the highest genetic distance. The two allexivirus isolates from Ethiopia (Et10 and Et20, GenBank accession number MH794550 and MH794555) identified as GarV-D in BLASTn analysis based on nucleotide sequences appeared to form an independent clade ancestral to GarV-B and GarV-X isolates being grouped far away from GarV-D isolates in the database. This was unexpected and further confirmation by analyzing more parts of the genome sequence will shade light on its phylogenetic and taxonomic grouping.

**Fig. 2:**
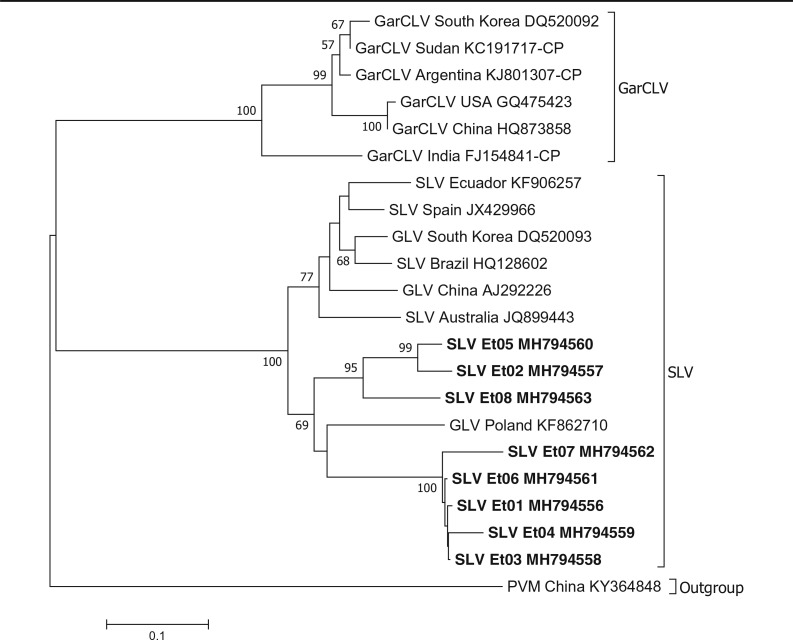
Phylogenetic tree based on nucleotide sequences showing the relationship of the different carlavirus isolates identified in this study with selected *Allium* carlavirus sequences in the GenBank. Potato virus M (PVM) is used as an outgroup. Only bootstrap values greater than 50% are shown and the scale bar indicates substitutions per site. Isolates from this study are highlighted in bold

In our analysis, sequences of SLV isolates from different parts of the world were grouped into two clusters. All Ethiopian SLV sequences were included in one of the groups, together with GLV isolate from Poland (KF862710) which formed a terminal branch supported with moderate to high bootstrap value (Fig. 2). The second group consisted of SLV isolates from various countries namely Australia, Ecuador, Spain, South Korea, Brazil and China. No Ethiopian isolate clustered with this group.

**Fig. 3:**
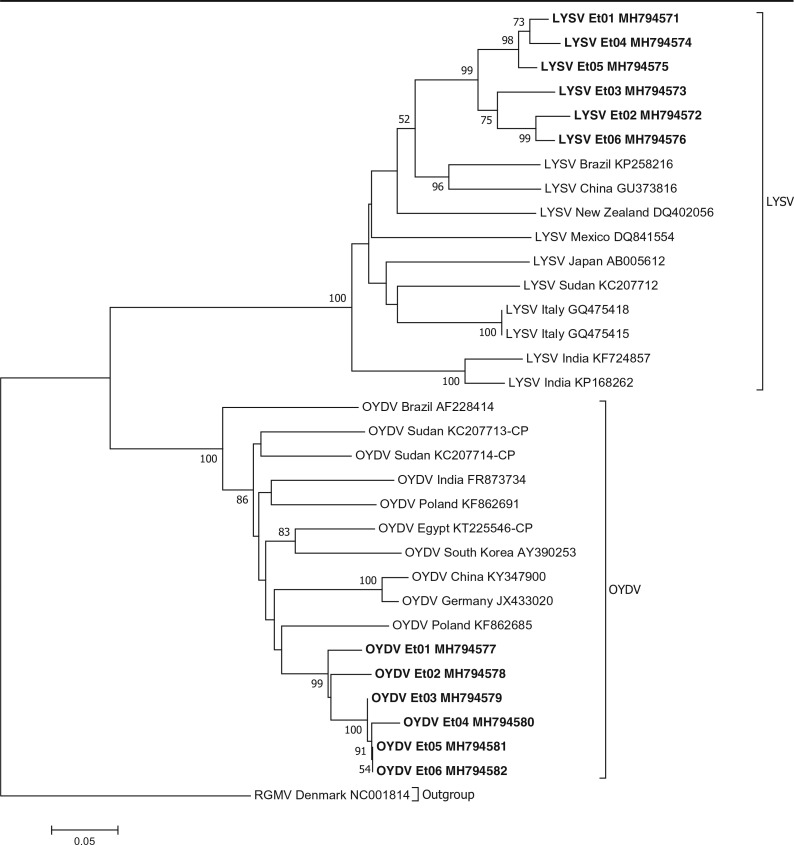
Phylogenetic tree based on nucleotide sequences showing the relationship of the different potyviruses isolates identified in this study with selected *Allium* potyvirus sequences in the GenBank. Ryegrass mosaic virus (RGMV) is used as an outgroup. Only bootstrap values greater than 50% are shown and the scale bar indicates substitutions per site. Isolates from this study are highlighted in bold

**Fig. 4:**
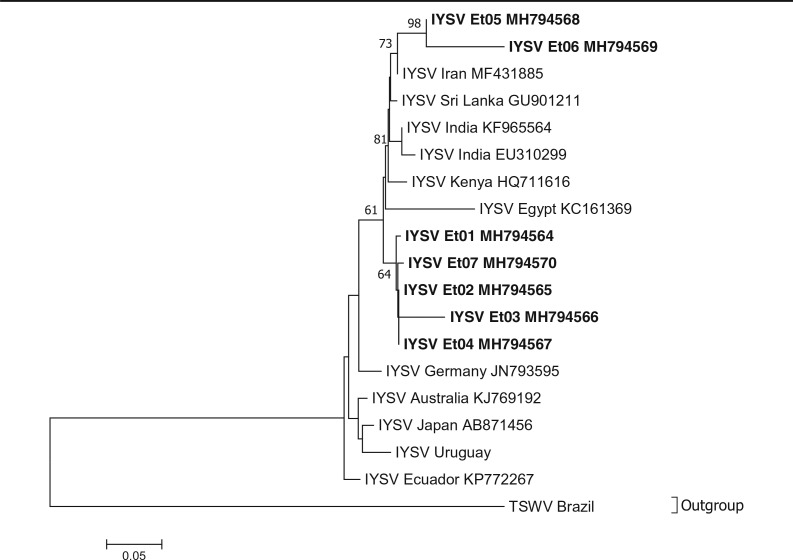
Phylogenetic tree based on nucleotide sequences showing the relationship of the different Iris yellow spot virus (IYSV) isolates identified in this study with other IYSV sequences in the GenBank. Tomato spotted wilt virus (TSWV) is used as an outgroup. Only bootstrap values greater than 50% are shown and the scale bar indicates substitutions per site. Isolates from this study are highlighted in bold

Phylogenetic analysis of garlic potyviruses from Ethiopia revealed that both OYDV and LYSV isolates from Ethiopia form distinct terminal branches (Fig. 3). All OYDV isolate from Ethiopia grouped at the terminal end of the phylogenetic tree making a distinct cluster with an OYDV isolate from Poland (KF862685) being genetically the closest. Isolates from Sudan and Egypt, countries geographically closest to Ethiopia, branched far away. Similarly, the LYSV isolates from Ethiopia grouped themselves in one cluster as divergent from isolate s from Brazil (KP258 216) and China (GU373816) which have similar genetic distance. Finally, all IYSV isolates from Ethiopia grouped themselves together with other IYSV isolates from India (EU310299 and KF965564), Sri Lanka (GU901211), Kenya (HQ711616), Iran (MF431885) and Egypt (KC161369) whereas isolates from Germany, Australia, Japan, Uruguay and Ecuador made a second group (Fig. 4).

PASC analysis carried out for the different garlic virus isolates sequenced from Ethiopia and other isolates from NCBI (data not shown) indicated that our isolates showed close similarity to species in the database and fulfill the species demarcation criteria suggested by ICTV and hence confirmed identification based on BLAST and phylogenetic analysis. The analysis also revealed that garlic virus isolates of the same species sequenced from Ethiopia showed close sequence similarity to each other which is also consistent with results of phylogenetic analysis. Recombination analysis on RDP4 revealed that no potential recombination event/s happened between isolates of the different virus groups of garlic from Ethiopia with their respective isolates from NCBI.

## Discussion

Garlic samples collected from most (75%) of farmers’ fields in Ethiopia were found to be infected with at least one virus belonging to either potyvirus, allexivirus, carlavirus or tospovirus groups. A total of seven viruses known to be prevalent on garlic elsewhere to a different degree were identified in Ethiopia. Of the seven distinct viruses reported, OYDV, LYSV and Garlic virus C have been previously reported from Ethiopia (Jemal et al. 2015). This is the first report of Garlic viruses-X, −D, IYSV and SLV. This work therefore considerably extends our knowledge on the diversity of garlic infecting viruses in Ethiopia and Africa at large. It also provides unprecedented insight into the evolutionary relationship of the Ethiopian garlic virus isolates to those in other countries. In addition, viruses were identified consistently from both symptomatic and asymptomatic garlic plants indicating that latent infection is common. Hence, recording based only on symptoms is unreliable indicator of virus incidence and employing laboratory based identification using RT-PCR and sequencing was mandatory for accurate virus identification.

The phylogenetic analyses carried out for allexiviruses, potyviruses, SLV and IYSV gave an insight into their relatedness to those in other countries. Ethiopian OYDV and LYSV may have been introduced to the country from Asia or Europe. The same evolutionary trend may have been followed by GarV-X. The data suggests that at least two groups of GarV-C isolates are found in Ethiopia most likely introduced from different sources (Fig. 1). SLV and IYSV may have also been introduced to Ethiopia from Europe or Asia. Overall, phylogenetic studies showed that Ethiopian garlic virus isolates are in most cases clustered together and their geographical origin and phylogenetic relatedness are often not correlated. Several studies conducted in other countries indicated similar evolutionary phenomena and attributed it to the fact that international exchange of vegetative planting materials between countries contributing to high diversity and rapid evolution of garlic viruses (Bereda et al. 2017; Koo et al. 2002; Mohammed et al. 2013; Taglienti et al. 2018; Wylie et al. 2012). Historically, Ethiopia has been actively involved in trade and germplasm exchange of horticultural spices including *Allium* crops with Asian, Middle East, European and North African countries. It is likely that such international exchange of non-tested *Allium* planting materials resulted in multiple introduction of viruses from various sources which evolved over time in geographical isolation in Ethiopia to distinct clades.

The presence of a complex mixture of viruses from several genera in high incidence in Ethiopian garlic crops is a cause for a serious concern in economic terms. The high incidence of virus detected in the laboratory analysis from both symptomatic and asymptomatic samples in farmers’ garlic fields is expected to cause a considerable reduction in yield and quality. The different viruses identified are expected to cause varying level of damage in the field. Among the two potyviruses, OYDV is the most common compared to LYSV being detected in 33 of the 40 potyvirus positive samples (82.5%) suggesting its economic importance in garlic production. Hence, future disease management efforts should target this virus. It is known that in single infection, OYDV and IYSV can cause severe symptoms in garlic compared to carlaviruses and allexiviruses which individually cause little or no disease symptoms. On the other hand, mixed infection of any of these viruses is reported to cause more severe diseases resulting in drastic reductions in yield (Cafrune et al. 2006; Lot et al. 1998; Paludan 1980). Due to the high level of contamination of garlic germplasm with viruses as revealed by this study, it is highly likely that the viruses can be disseminated from research centers to farmers’ fields when the germplasm is distributed to growers as improved cultivars.

The high incidence of IYSV in garlic production in Ethiopia poses special concern. This virus, transmitted by *Thrips tabaci* is first reported as a serious pathogen of onion in the Pacific Northwest (Gent et al. 2006), and later as infecting garlic in many countries. In Africa, it is reported in garlic from Egypt and Zimbabwe (Karavina et al. 2016). A study in Israel showed that incidence of IYSV was strongly correlated to the population of *Thrips tabaci* (Kritzman et al. 2001). *Thrips tabaci* is a common pest infesting *Allium* crops in Ethiopia. Further information on the distribution IYSV and its strains in Ethiopia, its other hosts including onion, the accurate identification of its thrips vector and studying its biology would be pursued to provide valuable information to help growers to control this devastating virus disease.

Our analysis did not indicate recombination event in all of the different Ethiopian garlic virus isolates we tested suggesting that none of the viruses are recombinant at least in the part of the genome analyzed. However, it should be noted that sequences for the different viruses infecting garlic generated in this study represent only small part from the viral genome providing us with a smaller chance for detecting potential recombination event compared to the whole virus genome. It would thus be more informative to conduct recombination analysis after generating full length sequences of Ethiopian garlic viruses in the future.

In conclusion, our work on garlic viruses in farmers’ fields and national germplasm collection provided unprecedented information on the relative incidence, distribution and diversity of viruses in the country and an insight into their evolutionary relationship, as well as their potential to cause damage singly or in mixed infection. The molecular assays used in this study for the identification of garlic viruses are proved to be highly sensitive and reliable. The degenerate carlavirus primer pair (Tsuneyoshi et al. 1998b) however did not amplify any carlavirus RNA from our samples including from those positive for SLV possibly due to the existence of sequence variation. In addition, specific primer pairs targeting viruses such as SYSV and GarCLV did not yield expected amplicons. Whatever the reasons for this may be, it is possible that there are viruses in garlic fields which could not be identified and reported in this work. It is also possible that the degenerate allexivirus and potyvirus primers may have preferentially detected some virus sequences over the others due to amplification bias. The use of primers sets amplifying garlic viruses not reported in this study and sequence independent methods such as next generation sequencing can reveal if there exist other unidentified and less common viruses infecting Ethiopian garlic crops.
