# Supplementary material for: Molecular identification, incidence and phylogenetic analysis of seven viruses infecting garlic in Ethiopia
Source: Eur J Plant Pathol. 2019 May 15;155:181–91. doi: 10.1007/s10658-019-01760-9 (PMC7680954; doi:10.1007/s10658-019-01760-9)
Supplement: Supplementary file 1 [file EJPP-155-181-s001.docx]

Table S1.

| **Zone** | **Location / Accession number** | **Potyvirus** | **Allexivirus** | **SLV** | **IYSV** |
| --- | --- | --- | --- | --- | --- |
| East shewa | Cheffee 19.1 | + | + | + |  |
|  | Cheffee 19.3 |  |  |  |  |
|  | Cheffee 22.2 | + | + | + |  |
|  | Cheffee 20.1 | + | + |  |  |
|  | Cheffee 20.3 | + | + |  |  |
| West shewa | Holetta 2 | + |  |  |  |
|  | Addis alem 15-135 |  |  |  |  |
|  | Sadamo west shewa |  |  | + |  |
| Arsi | Arsi Robe |  |  |  | + |
|  | Ticho 12(7) |  |  |  |  |
|  | Ticho 285 |  |  |  |  |
| East Gojam | Yelwa 4 | + | + |  |  |
|  | Yelwa 2.2 | + | + | + |  |
|  | Yelwa 5 | + |  | + |  |
|  | Yelwa 1.1 |  |  | + |  |
| West Gojam | Koga 3 | + |  |  |  |
|  | Bure 1.1 | + |  |  |  |
|  | Koga 1.2 |  |  |  |  |
|  | Bure 1.2 | + |  | + |  |
|  | Dembecha 2 |  |  | + |  |
|  | Koga 9 | + | + | + |  |
|  | Koga 1.1 | + | + |  |  |
|  | Koga 1 | + |  | + |  |
|  | Bure 1.1 |  |  | + |  |
| South Gonder | Arno 5 | + |  | + |  |
|  | Gumara 7 |  |  | + | + |
|  | Addis zemen | + | + | + |  |
|  | Awramba 5 | + | + |  |  |
|  | Awramba 5.1 |  |  |  |  |
|  | Addiszemen10 | + |  |  |  |
|  | Arno 8 | + | + |  |  |
|  | Arno 7 |  |  |  | + |
|  | Gumara 5 | + |  | + |  |
|  | Addis zemen 7 | + | + |  |  |
|  | Addis zemen 8 |  |  | + |  |
|  | Gumara 1 |  |  |  |  |
|  | Addis zemen 1.5 |  |  |  |  |
| North Gonder | Siemen park | + | + | + |  |
|  | Siemen park | + |  | + |  |
| Oromiya Liyu | Sendafa | + |  |  | + |
|  | Legedadi 26.1 |  |  |  |  |
|  | Mada Gudina |  |  |  |  |
|  | Legedadi 26.4 | + | + | + |  |
|  | Legedadi 26.5 |  |  |  |  |
| **Germplasm collection** | Ac 19/95 |  |  |  |  |
|  | Ac 68/DZ |  |  | + |  |
|  | Ac 30/03 | + |  |  |  |
|  | Ac 25-2/94 |  |  |  |  |
|  | Ac 21-1/94 |  |  |  |  |
|  | Ac 82-2/94 |  |  |  |  |
|  | Ac 88-1/94 |  |  |  |  |
|  | Ac 122-2/94 | + | + |  |  |
|  | Ac 35-2/94 |  |  |  |  |
|  | Ac 94-1/94 |  |  |  |  |
|  | Tseday | + | + |  |  |
|  | Ac 79/03 |  | + |  |  |
|  | Ac 53/03 |  |  |  |  |
|  | Ac 44/03 |  |  |  |  |
|  | Ac 56/03 |  |  |  |  |
|  | Ac 38/03 |  |  |  |  |
|  | Ac 07/03 | + | + | + |  |
|  | Ac 14/03 |  |  |  |  |
|  | Ac 44/03 | + | + | + |  |
|  | Ac 21/03 | + | + | + |  |
|  | Ac 03/03 |  |  |  |  |
|  | Ac 27/03 |  | + |  |  |
|  | Ac 74/03 | + | + |  |  |
|  | Ac 15/03 |  |  |  |  |
|  | Ac 62/03(DZ) |  |  | + |  |
|  | Ac 130-1/94 | + | + | + |  |
|  | Ac 32/03 |  | + | + |  |
|  | Ac 59/03 | + | + | + |  |
|  | Ac 29/03 |  |  |  | + |
|  | Ac 68/03 | + | + | + | + |
|  | Ac 70/03 |  | + |  |  |
|  | Ac 15-1/94 | + |  |  |  |
|  | Ac 46/03 | + |  |  |  |
|  | Ac11-1/94 | + | + |  |  |
|  | Ac 77/03 | + | + |  |  |
|  | Ac 66/03 |  | + |  |  |
|  | Ac22/03 |  |  |  | + |
|  | Ac 158-1/94 |  | + | + |  |
|  | Kurfitu | + | + | + |  |
|  | Ac 8-2/94 | + | + | + |  |
|  | Ac 76-1/94 |  |  | + |  |
|  | Ac 08/03 |  | + |  |  |
|  | Ac 57/03 | + | + | + |  |
|  | Ac76/03 |  | + | + |  |
|  | Ac 65/03 |  |  | + |  |
|  | Ac 10/03 |  | + | + |  |
|  | Ac 048/03 | + | + | + |  |
|  | Ac67/03 |  |  | + |  |
|  | Ac 75/03 | + | + |  |  |
|  | Holetta local | + | + |  |  |
|  | Ac 01/03 | + | + | + |  |
| **Total** | **95** | **46** | **41** | **39** | **7** |
